# Supplementary material for: The effects of genital myiasis on the diversity of the vaginal microbiota in female Bactrian camels
Source: BMC Vet Res. 2022 Mar 5;18:87. doi: 10.1186/s12917-022-03189-5 (PMC8897907; doi:10.1186/s12917-022-03189-5)
Supplement: Supplementary file 5 — Additional file 5. [file 12917_2022_3189_MOESM5_ESM.zip › MPL201709200_16s_yy/Treat1/css/js_css/jsPDF-master.zip!/jsPDF-master/docs/plugins_xmp_metadata.js.html]

plugins/xmp\_metadata.js - Documentation


## Home

### Classes

- jsPDF

### Global

- addFont
- addHTML
- addMetadata
- addPage
- autoPrint
- CapJoinStyles
- circle
- ellipse
- getFontList
- lines
- lstext
- output
- rect
- roundedRect
- save
- setDisplayMode
- setDrawColor
- setFillColor
- setFont
- setFontSize
- setFontStyle
- setLineCap
- setLineJoin
- setLineWidth
- setPage
- setProperties
- setTextColor
- text
- triangle

# plugins/xmp\_metadata.js

```
/** ==================================================================== 
 * jsPDF XMP metadata plugin
 * Copyright (c) 2016 Jussi Utunen, u-jussi@suomi24.fi
 * 
 * Permission is hereby granted, free of charge, to any person obtaining
 * a copy of this software and associated documentation files (the
 * "Software"), to deal in the Software without restriction, including
 * without limitation the rights to use, copy, modify, merge, publish,
 * distribute, sublicense, and/or sell copies of the Software, and to
 * permit persons to whom the Software is furnished to do so, subject to
 * the following conditions:
 * 
 * The above copyright notice and this permission notice shall be
 * included in all copies or substantial portions of the Software.
 * 
 * THE SOFTWARE IS PROVIDED "AS IS", WITHOUT WARRANTY OF ANY KIND,
 * EXPRESS OR IMPLIED, INCLUDING BUT NOT LIMITED TO THE WARRANTIES OF
 * MERCHANTABILITY, FITNESS FOR A PARTICULAR PURPOSE AND
 * NONINFRINGEMENT. IN NO EVENT SHALL THE AUTHORS OR COPYRIGHT HOLDERS BE
 * LIABLE FOR ANY CLAIM, DAMAGES OR OTHER LIABILITY, WHETHER IN AN ACTION
 * OF CONTRACT, TORT OR OTHERWISE, ARISING FROM, OUT OF OR IN CONNECTION
 * WITH THE SOFTWARE OR THE USE OR OTHER DEALINGS IN THE SOFTWARE.
 * ====================================================================
 */

/*global jsPDF */

/**
* Adds XMP formatted metadata to PDF
*
* @param {String} metadata The actual metadata to be added. The metadata shall be stored as XMP simple value. Note that if the metadata string contains XML markup characters "<", ">" or "&", those characters should be written using XML entities.
* @param {String} namespaceuri Sets the namespace URI for the metadata. Last character should be slash or hash.
* @function
* @returns {jsPDF}
* @methodOf jsPDF#
* @name addMetadata
*/

(function (jsPDFAPI) {
    'use strict';
    var xmpmetadata = "";
    var xmpnamespaceuri = "";
    var metadata_object_number = "";   
    
    jsPDFAPI.addMetadata = function (metadata,namespaceuri) {
        xmpnamespaceuri = namespaceuri || "http://jspdf.default.namespaceuri/"; //The namespace URI for an XMP name shall not be empty
        xmpmetadata = metadata;
        this.internal.events.subscribe(
            'postPutResources',
            function () {
                if(!xmpmetadata)
                  {
                  metadata_object_number = "";
                  }
                else
                  {
                  var xmpmeta_beginning = '<x:xmpmeta xmlns:x="adobe:ns:meta/">';
                  var rdf_beginning = '<rdf:RDF xmlns:rdf="http://www.w3.org/1999/02/22-rdf-syntax-ns#"><rdf:Description rdf:about="" xmlns:jspdf="' + xmpnamespaceuri + '"><jspdf:metadata>';
                  var rdf_ending = '</jspdf:metadata></rdf:Description></rdf:RDF>';
                  var xmpmeta_ending = '</x:xmpmeta>';
                  var utf8_xmpmeta_beginning = unescape(encodeURIComponent(xmpmeta_beginning));
                  var utf8_rdf_beginning = unescape(encodeURIComponent(rdf_beginning));
                  var utf8_metadata = unescape(encodeURIComponent(xmpmetadata));
                  var utf8_rdf_ending = unescape(encodeURIComponent(rdf_ending));
                  var utf8_xmpmeta_ending = unescape(encodeURIComponent(xmpmeta_ending));

                  var total_len = utf8_rdf_beginning.length + utf8_metadata.length + utf8_rdf_ending.length + utf8_xmpmeta_beginning.length + utf8_xmpmeta_ending.length;
                
                  metadata_object_number = this.internal.newObject();
                  this.internal.write('<< /Type /Metadata /Subtype /XML /Length ' + total_len + ' >>');
                  this.internal.write('stream');
                  this.internal.write(utf8_xmpmeta_beginning + utf8_rdf_beginning + utf8_metadata + utf8_rdf_ending + utf8_xmpmeta_ending);
                  this.internal.write('endstream');
                  this.internal.write('endobj');                
                  }
            }
        );
        this.internal.events.subscribe(
            'putCatalog',
            function () {
                if (metadata_object_number) {
                    this.internal.write('/Metadata ' + metadata_object_number + ' 0 R');
                }
            }
        );        
        return this;
    };
}(jsPDF.API));
```

  

Documentation generated by JSDoc 3.4.2 on Sun Oct 09 2016 11:08:27 GMT+0100 (BST) using the docdash theme.
